# Supplementary material for: Oxygraphy Versus Enzymology for the Biochemical Diagnosis of Primary Mitochondrial Disease
Source: Metabolites. 2019 Oct 10;9(10):220. doi: 10.3390/metabo9100220 (PMC6835216; doi:10.3390/metabo9100220)
Supplement: Supplementary file 1 [file metabolites-09-00220-s001.pdf]

Supplementary:

Table S1. Retrospective clinical diagnostic prediction scoring of genetically confirmed PMD patients. A score of ≥2 indicates the likeliness of a PMD, which is used clinically to initiate follow up of biochemical and genetic testing. Abbreviations: CC, corpus callosum; CSF: cerebrospinal fluid; EMG, electromyogram; FTT, failure to thrive; GI, gastrointestinal; MRS, magnetic resonance spectroscopy; WM: white matter.

		Muscle						Neurological									Multi system									Combined clinical		Metabolic and imaging															All
Identifier #	Mutated gene or complex	Myopathy	Abnormale EMG	Motor developmental delay	Exercise intolerance	Total	Maximum out of 2	Developmental delay	Speech delay	Dystonia	Ataxia	Spasticity	Neuropathy	Seizures or encephalopathy	Total	Maximum out of 2	GI tract	Growth delay or FTT	Endocrine	Immune	Eye & auditory	Renal tubular acidosis	Cardiomyopathy	Total	Maximum out of 3	Total	Maximum out of 4	Lactate	Alanine	Krebs cycle intermediates	Ethyl or methly malonic aciduria	3 methyl glutaconic acid	CSF lactate, alanine	Leigh like	Stroke like episodes	Lactate on MRS	Leukoencephalopathy with brain stem and spinal cord involvement	Cavitating leukoencephalopathy	Leukoencephalopathy with thalamus involvement	Deep cerebral WM involvement and CC agenesis	Total	Maximum out of 4	Maximum out of 8
45	*TMEM126B*	1	0	0	1	2	2	0	0	0	0	0	0	0	0	0	0	0	0	0	0	0	0	0	0	2	2	2	1	0	0	0	1	0	0	0	0	0	0	0	4	6	6
30	*ACAD9*	0	0	0	1	1	1	0	0	0	0	0	0	0	0	0	0	0	0	0	0	0	1	1	1	2	2	2	1	1	0	1	0	0	0	0	0	0	0	0	5	6	6
33	*MT-ND1*	0	0	1	0	1	1	1	1	0	0	0	0	1	3	2	0	1	0	0	0	0	1	2	2	5	4	2	1	1	0	0	1	0	0	0	0	0	0	0	5	8	8
48	*ND6*	0	0	0	1	1	1	0	0	1	0	1	1	1	4	2	0	0	0	0	1	0	0	1	1	4	4	0	0	0	0	0	1	2	0	0	0	0	0	0	3	7	7
2737	*NDUFS1*	0	0	1	0	1	1	1	1	0	0	1	0	1	4	2	0	1	0	0	0	0	0	1	1	4	4	2	0	1	0	0	1	2	0	0	0	0	0	0	6	8	8
2736	*NDUFS2*	0	0	0	0	0	0	0	0	0	0	1	0	1	2	2	0	0	0	0	0	0	0	0	0	2	2	0	0	0	0	0	1	2	0	1	0	0	0	0	4	6	6
2497	*NDUFA13* AND *PGM1*	0	0	1	0	1	1	1	1	1	0	1	0	1	5	2	0	1	0	0	1	0	0	2	2	5	4	2	1	0	0	0	1	2	0	1	0	0	0	0	5	8	8
52	*SURF1*	1	0	1	0	2	2	0	0	0	1	0	0	1	2	2	0	1	0	0	0	1	1	3	3	7	4	2	0	1	1	0	0	2	0	0	0	0	0	0	6	8	8
55	*SURF1*	0	0	1	0	1	1	1	1	0	1	1	0	1	5	2	1	1	0	0	0	1	0	3	3	6	4	2	0	1	0	0	1	2	0	0	0	0	0	0	6	8	8
2264	*MT-ATP6*	0	0	1	0	1	1	1	0	0	1	0	0	1	3	2	0	0	0	0	0	1	0	1	1	4	4	2	0	1	0	0	1	0	0	0	0	0	0	0	4	8	8
47	*AGK*	1	0	1	0	2	2	1	1	0	0	0	0	0	2	2	0	1	0	0	1	0	1	3	3	7	4	2	1	0	0	0	1	0	0	0	0	0	0	0	4	8	8
34	*EARS2*	0	0	1	0	1	1	1	1	0	1	0	0	0	3	2	0	0	0	0	0	0	0	0	0	3	3	2	0	0	0	0	1	0	0	1	0	0	0	0	4	7	7
43	*MRPL44*	1	0	0	1	2	2	0	0	0	1	0	0	1	2	2	1	0	0	1	1	0	1	4	3	7	4	0	0	0	0	0	0	0	0	0	1	0	0	0	1	5	5
42	Large mtDNA deletion	1	1	0	0	2	2	0	e	0	0	0	0	0	0	0	0	0	0	0	0	0	0	0	0	2	2	0	0	0	0	0	0	0	0	0	0	0	0	0	0	2	2
41	Large mtDNA deletion	1	1	0	0	2	2	0	0	0	0	0	0	0	0	0	0	0	0	0	1	0	1	2	2	4	4	0	0	0	1	1	0	0	0	0	0	0	0	0	2	6	6
50	*MT-TD*	1	1	0	1	3	2	0	0	0	0	0	0	0	0	0	0	0	0	0	0	0	0	0	0	2	2	2	1	1	0	0	0	0	0	0	0	0	0	0	4	6	6
36	*MT-TE*	0	0	1	1	2	2	1	1	0	0	0	1	0	3	2	0	1	1	0	0	0	0	2	2	6	4	0	0	0	0	0	0	0	0	0	0	0	0	0	0	4	4
57	*MT-TE*	1	0	1	1	3	2	0	0	0	0	0	0	0	0	0	0	0	1	0	0	0	0	1	1	3	3	2	1	0	0	1	0	0	0	0	0	0	0	0	4	7	7
58	*MT-TL1*	1	0	0	1	2	2	0	0	0	0	0	1	1	2	2	0	0	1	1	1	1	1	5	3	7	4	0	0	0	0	0	0	0	0	0	0	0	0	0	0	4	4
123	*MT-TL1*	1	0	1	1	3	2	0	0	0	0	0	0	0	0	0	0	1	0	0	0	0	1	2	2	4	4	2	1	0	0	0	0	0	0	0	0	0	0	0	3	7	7
53	*MT-TL1*	1	1	0	1	3	2	0	0	0	0	0	1	1	2	2	0	0	1	0	1	0	1	3	3	7	4	2	1	0	0	0	0	0	0	0	0	1	0	0	4	8	8
54	*MT-TL1*	1	1	0	1	3	2	0	0	0	0	0	0	1	1	1	0	0	0	0	1	0	1	2	2	5	4	2	0	0	0	0	0	0	0	0	0	0	0	0	2	6	6
72	*MT-TL1*	0	1	0	0	1	1	0	0	0	1	0	0	1	2	2	0	0	1	0	1	0	1	3	3	6	4	2	0	0	0	0	1	0	2	0	0	0	1	0	6	8	8
40	*MT-TL1*	0	1	0	0	1	1	0	0	0	0	0	0	0	0	0	0	1	1	0	1	1	1	5	3	4	4	0	0	0	0	0	0	0	0	0	0	0	0	0	0	4	4
51	*MT-TN*	1	0	0	0	1	1	1	0	0	0	1	0	1	3	2	0	1	1	0	0	1	0	3	3	6	4	2	0	0	0	0	1	0	0	0	1	0	0	0	4	8	8
124	*TWNK*	1	1	0	0	2	2	0	0	0	0	0	1	0	1	1	0	0	0	0	0	0	0	0	0	3	3	0	0	0	0	0	0	0	0	0	0	0	0	0	0	3	3
35	*POLG*	0	0	0	0	0	0	0	0	0	1	1	0	0	2	2	1	1	0	0	0	0	0	2	2	4	4	0	0	1	1	0	0	0	0	0	1	0	0	0	3	7	7
38	*POLG*	0	0	0	0	0	0	0	0	0	0	0	0	1	1	1	1	1	0	1	0	0	0	3	3	4	4	2	0	0	0	0	0	0	0	0	0	1	0	0	3	7	7
120	*POLG*	1	1	0	1	3	2	0	0	0	0	0	0	0	0	0	0	0	0	0	0	0	0	0	0	2	2	0	0	0	1	0	0	0	0	0	0	0	0	0	1	3	3
59	*ATAD3*	0	0	1	0	1	1	1	1	0	0	1	1	0	4	2	0	1	0	1	0	0	0	2	2	5	4	0	0	1	0	1	0	0	0	0	0	0	0	0	2	6	6
2130	*PDHA1*	0	0	1	0	1	1	1	1	1	0	1	0	1	5	2	0	0	0	0	0	0	0	0	0	3	3	2	1	0	0	0	0	0	0	0	0	0	0	0	3	6	6
31	*PDHA1*	0	0	1	0	1	1	1	1	0	0	0	0	1	3	2	1	0	0	0	1	0	0	2	2	5	4	2	1	1	0	0	1	0	0	0	0	0	0	1	6	8	8
128	*SLC25A42*	1	1	0	1	3	2	0	0	0	0	0	0	0	0	0	1	0	0	0	0	0	1	2	2	4	4	2	1	0	0	0	0	0	0	0	0	0	0	0	3	7	7
2738	*SLC25A42*	1	0	1	0	2	2	0	1	0	1	1	1	0	4	2		0	1	0	0	0	0	1	1	5	4	2	0	0	0	0	0	0	0	0	0	0	0	0	2	6	6

0 — Highest

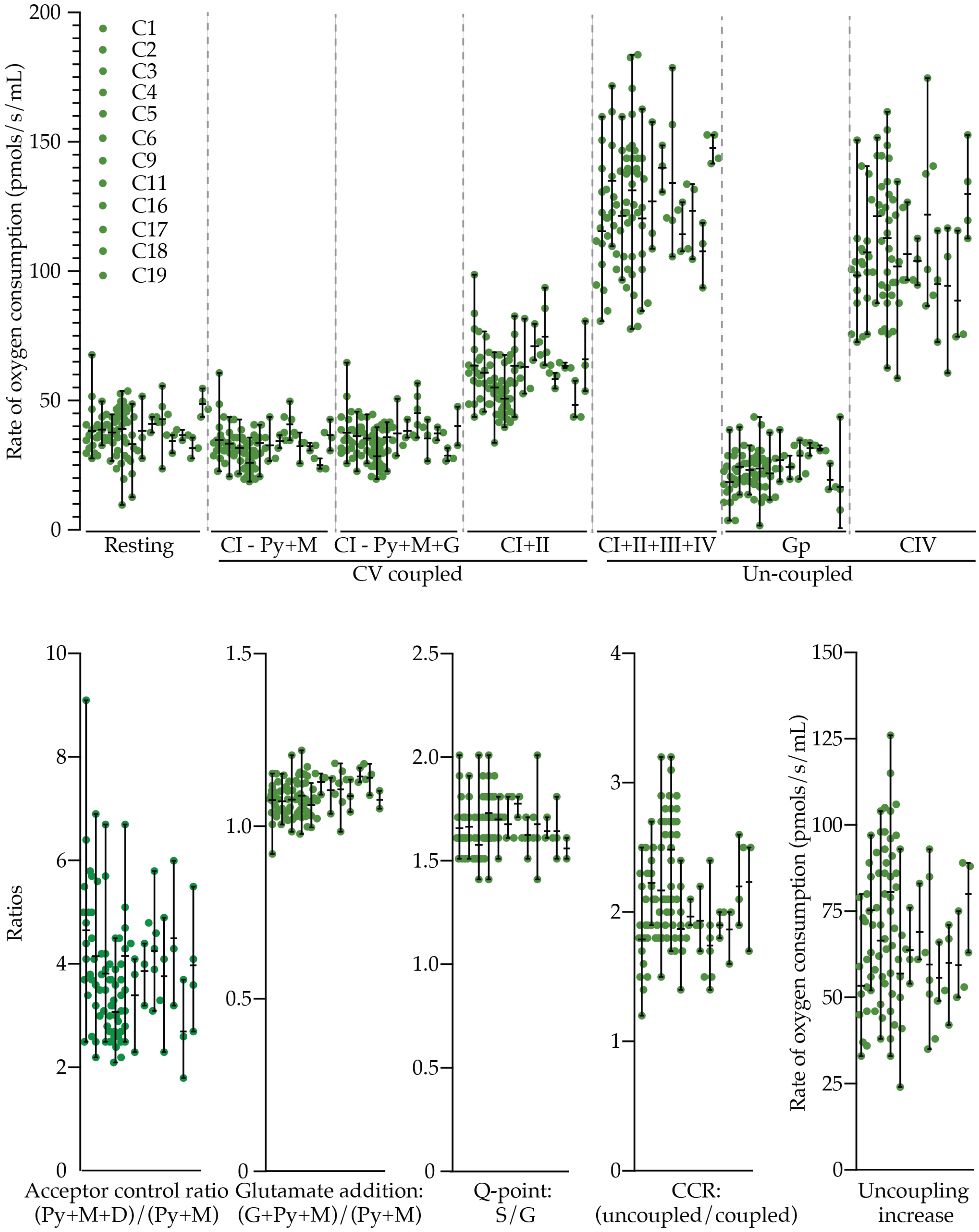

Figure S1. Technical replicates for oxygraphy testing in control fibroblasts cells. Resting, coupled, uncoupled rates of respiration and derived values. Mean is displayed for each control, and error bars show range of technical replicates, Abbreviations: C1-19, controls 1-19; CI-V, OXPHOS complexes I-V; CCR, coupling control ratio; Gp, glycerophosphate; G, glutamate; M, malate; Py, pyruvate; S, succinate.

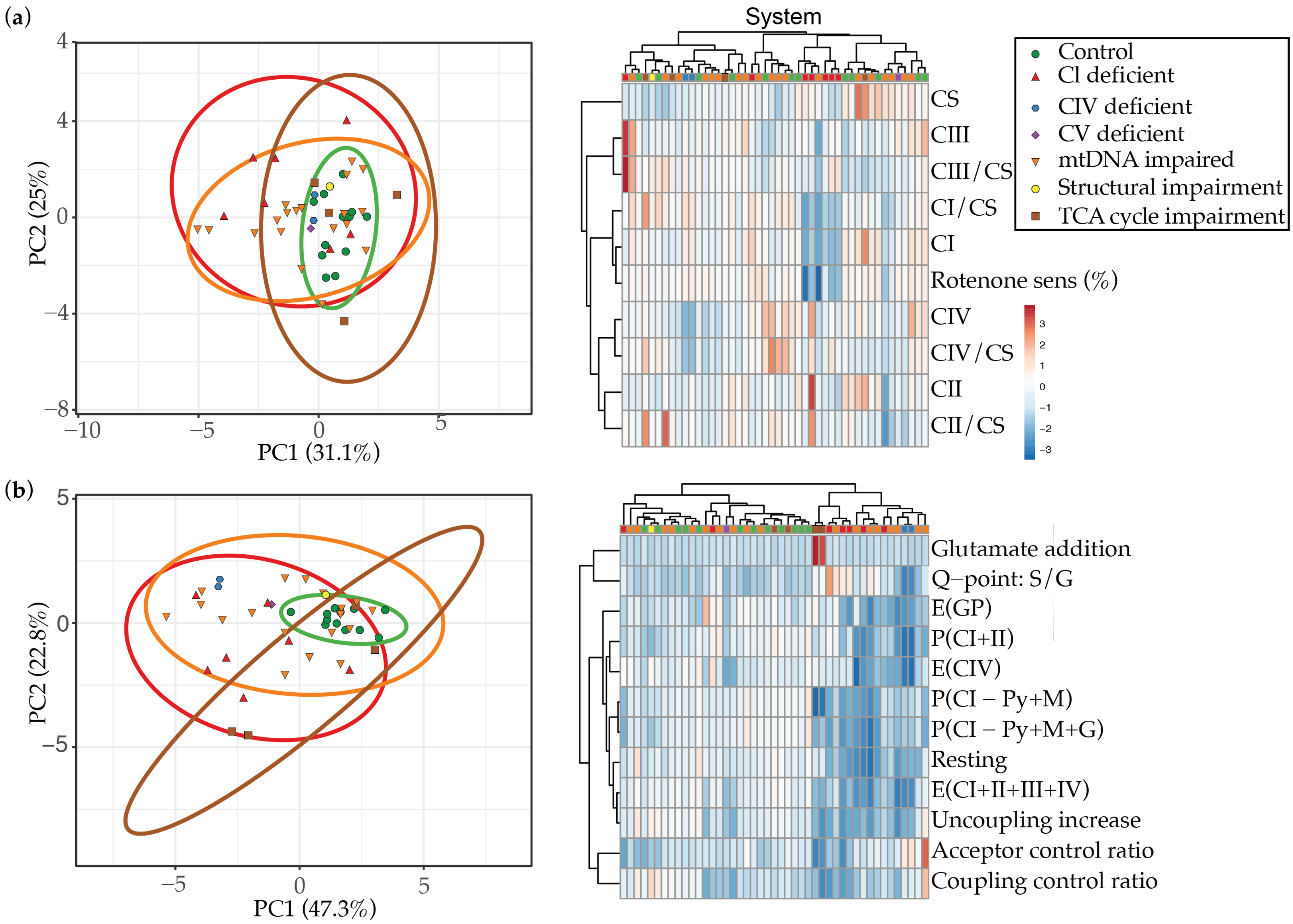
(a)
PC2 (25%)
PC1 (31.1%)
System
CS
CIII
CIII/CS
CI/CS
CI
Rotenone sens (%)
CIV
CIV/CS
CII
CII/CS
Control
CI deficient
CIV deficient
CV deficient
mtDNA impaired
Structural impairment
TCA cycle impairment
(b)
PC2 (22.8%)
PC1 (47.3%)
Glutamate addition
Q–point: S/G
E(GP)
P(CI+II)
E(CIV)
P(CI – Py+M)
P(CI – Py+M+G)
Resting
E(CI+II+III+IV)
Uncoupling increase
Acceptor control ratio
Coupling control ratio

Figure S2. Principal component and heat map analysis of enzymology and oxygraphy results. (**a**) Enzymology and (**b**) oxygraphy results were analysed using the ClustVis tool [1], and presented here as PCA plots and heat maps. For PCA plots, unit variance scaling is applied to rows; SVD with imputation is used to calculate principal components. X and Y axis show principal component 1 and principal component 2 that explain (a) 31.3% and 25% and (b) 47.3% and 22.8% of the total variance, respectively. Prediction ellipses are such that with probability 0.95, a new observation from the same group will fall inside the ellipse. For heat maps, rows are centred; unit variance scaling is applied to rows. Imputation is used for missing value estimation. Both rows and columns are clustered using correlation distance and average linkage.

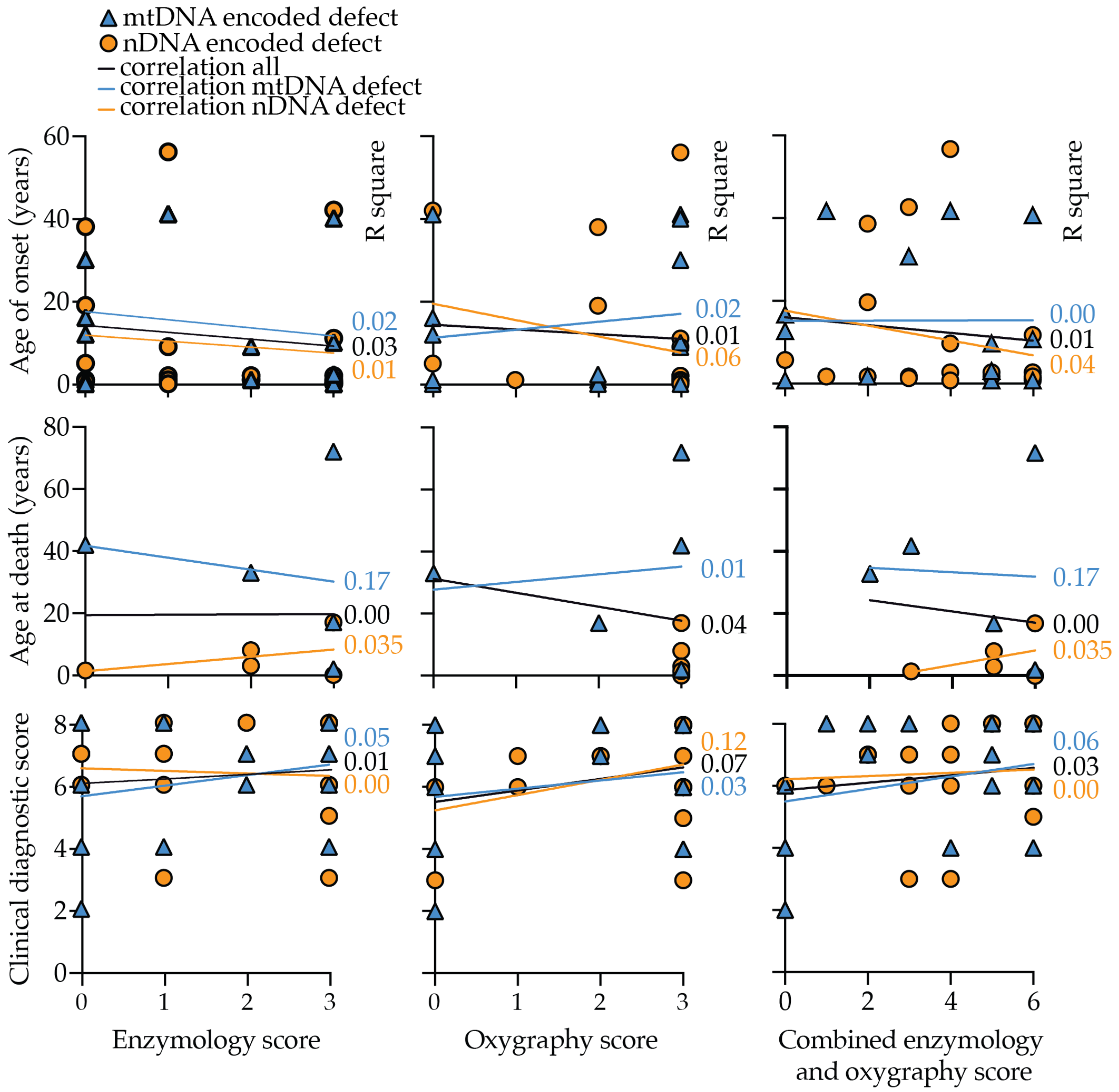

Figure S3. Biochemical correlations with disease characteristics. The disease predictions made through the enzymological and oxygraphy methods on PMD patient fibroblasts (subdivided into mtDNA or nDNA encoded mutations) based on the Z scores (see materials and methods) were converted to scores: Unlikely, 0; possible, 1; likely, 2; and very likely, 3. These were then correlated against the age of onset of disease, the clinical diagnostic score or the age of death (if applicable). R square values for correlations are displayed as generated in Prism 8.

References

1. Metsalu, T.; Vilo, J. ClustVis: a web tool for visualizing clustering of multivariate data using Principal Component Analysis and heatmap. *Nucleic Acids Res* **2015**, *43*, W566-570, doi:10.1093/nar/gkv468.
